# The detection of axillary lymph node metastases from breast cancer by radiolabelled monoclonal antibodies: a prospective study.

**DOI:** 10.1038/bjc.1989.61

**Published:** 1989-02

**Authors:** J. J. Tjandra, N. P. Sacks, C. H. Thompson, M. J. Leyden, S. A. Stacker, M. Lichtenstein, I. S. Russell, J. P. Collins, J. T. Andrews, G. A. Pietersz

**Affiliations:** Department of Pathology, University of Melbourne, Parkville, Victoria, Australia.

## Abstract

**Images:**


Br. J. Cancer (1989), 59, 296–302 © The Macmillan Press Ltd., 1989

### The detection of axillary lymph node metastases from breast cancer by radiolabelled monoclonal antibodies: a prospective study

J.J. Tjandra[1,2], N.P.M. Sacks[1], C.H. Thompson[1], M.J. Leyden[3], S.A. Stacker[1], M. Lichtenstein[2], I.S. Russell[2], J.P. Collins[2], J.T. Andrews[2], G.A. Pietersz[1] & I.F.C. McKenzie[1]

[1]*Research Centre for Cancer and Transplantation, Department of Pathology, The University of Melbourne, Parkville, Victoria 3052, Australia;* [2]*Royal Melbourne Hospital, Parkville, Victoria 3050, Australia and* [3]*St Andrew's Hospital, Claredon Place, East Melbourne, Victoria 3002, Australia.*

**Summary** In a prospective study to assess the accuracy of monoclonal immunoscintigraphy for the detection of axillary lymph node metastases in breast cancer, two murine monoclonal antibodies that react with human breast cancer (3E1.2 and RCC-1) were labelled with $^{131}$iodine, and the radiolabelled antibody was injected subcutaneously into the interdigital spaces of both hands of 40 patients, 36 of whom had breast cancer and the remaining four of whom had fibroadenoma (the normal, contralateral axilla was used as a control). Of the patients with breast cancer, the findings from the scintigraphy images were correlated with histopathology or cytology of the axillary lymph nodes; images were regarded as positive and hence indicative of lymph node metastases if the amount of background-subtracted radioactive count in axilla on the side of breast cancer exceeded the contralateral normal side by a ratio $\geqslant 1.5:1.0$ as assessed by computer analysis. Using this method, immunoscintigraphy had an overall sensitivity of 33% (23% with $^{131}$I-3E1.2 and 50% with $^{131}$I-RCC-1) for the detection of lymph node metastases and a specificity of 63% (67% with $^{131}$I-3E1.2 and 60% with $^{131}$I-RCC-1) with problems of non-specific uptake by presumably normal lymph nodes. The results of immunoscintigraphy obtained with $^{131}$I-RCC-1 (IgG) were superior to $^{131}$I-3E1.2 (IgM) although the accuracy of immunoscintigraphy using $^{131}$I-RCC-1 (56%) was not much better than preoperative clinical assessment (50%). However, there were cases when immunoscintigraphy using radiolabelled antibody (IgM or IgG) detected axillary lymph node metastases not suspected by clinical examination. Thus it appears that while immunoscintigraphy may be a useful adjunct to preoperative clinical assessment and is simple and safe, a major improvement in its accuracy is needed before it can replace axillary dissection and histological examination in the accurate staging of axilla in breast cancer.

The detection of overt tumour deposits by means of gamma-camera imaging and radiolabelled monoclonal antibodies to tumour-associated antigens (immunoscintigraphy) has met with encouraging results (Armitage *et al.*, 1984; Epenetos, 1985; Leyden *et al.*, 1986; Mach *et al.*, 1981; Rainsbury, 1984; Smedley *et al.*, 1983; William *et al.*, 1984). However, most of these studies have been performed on patients with well documented and widespread disease; few being prospective studies of the value of monoclonal immunoscintigraphy in the initial staging of patients with malignant disease. Immunoscintigraphy is complicated in that imaging using intravenously administered radiolabelled antibodies has a considerable background radioactivity because of the uptake of radiolabelled material in the blood pool and extravascular spaces, and antibodies may be catabolised before reaching their target, resulting in only a very small tumour uptake. In addition, dehalogenation of radiolabelled antibody, poor penetration of the conjugate into tumour deposits and antibody binding to cross-reactive antigens present on normal cells are further limitations to successful antibody targetting and therefore of immunoscintigraphy (Bradwell *et al.*, 1985; Rockoff *et al.*, 1980).

It is possible that intra-lymphatic delivery of antibody may avoid some of these problems. Several investigators have used subcutaneously injected radiolabelled anti-tumour antibodies to delineate metastatic deposits in regional lymph nodes (DeLand *et al.*, 1979; Thompson *et al.*, 1984) following the demonstration, in animals, that this route of administration permits more efficient delivery (>20% of injected dose) to lymphoid target cells in regional lymph nodes and avoids background uptake in other sites (Weinstein *et al.* 1982, 1984). It is suggested that this high level of antibody uptake into tumour-containing lymph nodes was due to the presence of an intact basement membrane about blood vessels, and the absence of one about lymphatics, allowing the preferential uptake to the lymphatics (Bergvist *et al.*, 1983; Leak, 1971; Weinstein *et al.*, 1983, 1984). Thompson *et al.* (1984) reported a preliminary study of eight patients with breast cancer using subcutaneously administered $^{131}$I-labelled anti-breast cancer antibody (3E1.2) and showed accurate localisation of tumour deposits in axillary lymph nodes in seven axillae with palpable lymph nodes and in two axillae with impalpable lymph nodes. On this basis, a prospective study in a larger number of patients presenting with suspected breast cancer was performed using the same techniques.

### Materials and methods

### *Monoclonal antibodies*

The murine monoclonal anti-breast antibodies (MoAbs) used were 3E1.2 (IgM) and RCC-1 (IgG 2a, formerly called 17.1), (Stacker *et al.*, 1985; Thompson *et al.*, 1983). By immunoperoxidase staining both 3E1.2 and RCC-1 antibodies react strongly with the membrane and cytoplasm of breast carcinomas in about 90% of cases and have only minimal reaction with normal breast tissue and with the tissues of relevance to this study (e.g. muscle, fat, endothelium, lymph nodes or erythrocytes). The monoclonal antibody 3E1.2 was raised against fresh human breast carcinoma (Stacker *et al.*, 1985) and was obtained in the ascites form. Purification of the 3E1.2 antibody from ascites fluid was achieved by treatment with Freon ($ClCF_2$,$CCl_2F$; Aldrich Chemical Co., Milwaukee, WI) to remove lipid, then dialysis against 5 mM Tris-HCl pH 7.5 and the precipitate was resuspended in 20 mM borate buffer pH 8.0, 0.3 M NaCl. The monoclonal antibody RCC-1 was produced by immunising inbred Biozzi mice with the MCF-7 breast cell line (Thompson *et al.*, 1983) and was isolated from ascitic fluid by precipitation with 40% ammonium sulphate, then purified by adsorption onto

Correspondence: I.F.C. McKenzie.
Received 20 June 1988, and in revised form, 4 October 1988.

Protein A-Sepharose (Pharmacia Inc., Piscataway, NJ) and eluting with 0.2 M glycine-HCl (pH 2.8).

The antibody activity was determined either by a rosetting technique (Parish & McKenzie, 1978) or by the immunoperoxidase method on sections of breast carcinoma and the purity tested by sodium dodecyl sulphate polyacrylamide gel electrophoresis (SDS-PAGE). The purified antibodies were filtered through a 0.22 $\mu$m Millex-GV filter (Millipore, Bedford, Ann Arbor, MI) and tested for pyrogens and sterility before and after radiolabelling (Pharmacology Department, Melbourne University and Sigma Pharmaceuticals, Clayton, Victoria, Australia).

*Iodination of monoclonal antibodies*

On the day of injection, purified 3E1.2 antibody or RCC-1 antibody (50–200 $\mu$g) was labelled with 5 mCi of $^{131}$I to a specific activity of 1–5 mCi of $^{131}$I per mg of antibody by means of iodobead (Markwell, 1982) or chloramine T method (Greenwood *et al.*, 1963) and the $^{131}$I-labelled antibody was separated from free iodine on a Sephadex G-25 column (PD10, Pharmacia, Sweden). The radioactivity of the iodinated antibody was measured in both a gamma counter (LKB Wallac 1260, Finland) and a radioisotope dose calibrator (Capintec CRC-2Ni, Capintec, New York) and the radiolabelled protein peak pooled. The sample was centrifuged at 100,000 *g* for 60 min to remove aggregated proteins and filtered through a 0.22 $\mu$m Millipore filter in a sterile laminar-flow hood. The immunoreactivity of the antibody was tested before and after radioiodination by rosetting techniques or by the immunoperoxidase staining as described above.

*Patients*

Forty patients with clinically suspected breast cancer were studied prospectively. Following histological or cytological examination, 36/40 patients had breast cancer (33 had stage I or II and three had stage IV breast cancer using the standard UICC classification) and 4/40 patients had benign breast disease (Bearhs & Myers, 1983). The radiolabelled antibody was injected subcutaneously in the inter-digital spaces of both hands; each patient also received potassium iodide (2 ml at 16.54% w/v) and sodium perchlorate (400 mg) orally 1 h before the injection to inhibit thyroid uptake of $^{131}$I; the potassium iodide was continued for 6 days after the injection. Of the patients studied, 55% (22/40) received $^{131}$I-3E1.2 antibody and the remainder $^{131}$I-RCC-1 antibody. Blood samples were obtained from 24/40 patients immediately before and 4 weeks after injection of radioiodinated monoclonal antibody for determining human anti-mouse antibody (HAMA) response. The clinical data were not made available until completion of the study.

*Imaging*

About 16–24 h after injection of the radiolabelled antibody, anterior scintiphotos of chest and both axillae were obtained with a Toshiba GCA402 gamma-camera using a high-energy parallel hole collimator and computerised acquisition with an Informatek Simis 4 computer. A window setting of 360 keV with a 20% window was used, images were obtained over a period of 600 seconds and then digitally recorded into a matrix of 128 × 128 words. Regions of interest in the images were defined by manual drawing by two independent observers over the axillary lymph node regions on both sides using anatomical landmarks as well as adjacent background and has been found to be remarkably reproducible. The data were decay-corrected and the fraction of radiolabelled antibody localised in the axillary nodes (*F*) was estimated by measurement of nodal uptake (*N*) with the gamma-camera, and compared with uptake in the other regions of interest and the amount of radiolabelled antibody injected (*I*). Nodal uptake was adjusted for background activity (*b*), camera response and attenuation through the anterior axillary fold using an attenuation factor (*A*) calculated using a known source placed in the axilla. The following formulae were used:

$$F(\%) = N_1/I \times 100 \qquad [1]$$

$$N_2(\text{c.p.m.}) = N_1(\mu\text{Ci}) \times \text{camera sensitivity} \qquad [2]$$

$$N_2 = \left[n - \left(\frac{bP_n}{P_b}\right)\right] \times \frac{A}{e^{-\mu x}}. \qquad [3]$$

where $F$ = fraction of radiolabelled antibody localised in the nodes; $N$ = nodal uptake ($N_1$ in $\mu$Ci, $N_2$ in c.p.m.); $n$ = total gamma camera counts (c.p.m.) over lymph node regions; $I$ = actual injected dose ($\mu$Ci); $b$ = background activity (c.p.m.); $P_n$ = number of pixels in region of interest (lymph node region); $P_b$ = number of pixels in background; $A$ = attenuation factor; $e^{-\mu x}$ = factor for isotope decay = 0.94 for $^{131}$I at 18 h; camera sensitivity (122.4 c.p.m. $\mu$Ci$^{-1}$) was determined by counting the amount of count per minute (c.p.m.) with known amount (1 $\mu$Ci) of $^{131}$I.

Studies were reported as positive and therefore indicative of lymph node metastases if the number of counts in the axilla on the tumour side exceeded the normal side by a ratio equal to or greater than 1.5:1.0, after adjustment for background activity as indicated above. The amount of nodal uptake of radioactivity in $\mu$Ci ($N_1$) was also calculated for each axilla using the above formulae and the mean nodal uptake in each axilla was expressed as the percentage (%) of the injected dose. A comparison of the mean nodal uptake between axillae with and without lymph node metastases can therefore be made.

*Analysis of excised axillary lymph nodes*

Each node was processed and 6 $\mu$m sections were stained with Haematoxylin and Eosin for histological examination. Immunoperoxidase staining was performed in some cases of snap-frozen sections (with RCC-1 antibody) or formalin fixed, paraffin embedded sections (with 3E1.2 antibody) (Thompson *et al.*, 1983; Tjandra *et al.*, 1988).

*Human anti-mouse antibody response*

Human antibodies against the murine MoAbs were measured by an enzyme linked immunosorbent assay (ELISA), modified from that previously described (Schroff *et al.*, 1985). Ninety-six-well flexible polyvinyl chloride plates (Costar, Cambridge, MA) were coated with 50 $\mu$l per well of administered MoAb (5 $\mu$g ml$^{-1}$ of purified 3E1.2 or RCC-1 MoAbs) in a 0.1 M carbonate buffer, pH 9.6 and incubated at 37°C for 2 h. The plates were then washed with PBS, 0.05% Tween and non-specific binding blocked with 1% bovine serum albumin, PBS pH 7.6 for 2 h at 37°C. Serial dilution of patients' sera and pooled normal human serum (50 $\mu$l per well) in PBS, 0.05% Tween 20 were performed. After washing the coated and blocked plate with PBS, 0.05% Tween 20, diluted serum samples (50 $\mu$l per well) were added to the coated wells and left for overnight incubation at 4°C. Plates were then washed with PBS, 0.05% Tween 20 and then reacted with 50 $\mu$l per well of phosphatase-labelled affinity purified goat anti-human immunoglobulin (Kirkegaard and Parry, MD) at 37°C for 3 h. The plates were then washed extensively with PBS, 0.05% Tween 20 and 50 $\mu$l per well of alkaline phosphatase substrate was added. The colour reaction was read with an ELISA plate reader (Titretek, Multiscan, MC) at a wavelength of 405 nm. Results were expressed as the absorbance value of patient serum compared with pooled normal human serum and a positive test was considered to be a value at least twice the control.

*Statistical analysis*

The data were analysed statistically using the $\chi^2$ test and $P<0.05$ was regarded as significant.

### Results

The investigations were performed essentially as outpatient procedures unless for special medical or social reasons relevant to the surgery when the patients were in hospital.

*Toxicity*

Antibody administration was well tolerated by all the patients with no adverse reactions occurring except for the development of human anti-mouse antibody response at 4 weeks after injection in 2/24 patients (patients 1 and 32), one of whom had $^{131}$I-3E1.2 and the other $^{131}$I-RCC-1.

*Immunoscintigraphy with $^{131}$I-3E1.2 (IgM)*

The results of the scans in 22 patients (patients 1–22) with suspected primary breast cancer are shown in Table I. These were correlated with histological or cytological examination of the axillary lymph nodes except in patients subsequently proved to have benign breast disease. Correct prediction of the axillary lymph node status was obtained in 41% (9/22) of the patients by the preoperative scans and in 59% (13/22) of the patients by preoperative clinical assessment (Table II). Thirteen of the 22 patients had histologically or cytologically proven axillary lymph node metastases: 3/13 (23%) patients were detected by the scan, and 7/13 (54%) patients by clinical examination. Nine of the 22 patients did not have lymph node metastases and a negative scan was obtained in 6/9 (67%) patients; in a comparable number (6/9 or 67%), the axillae were considered not involved by metastases on clinical examination (Table I).

**Table I** Clinical, histology and immunoscintigraphy data in patients with suspected breast cancer

| *Patient* | *Side of tumour* | *Histology*[a] | *Axillary*[b] *nodal pathology* | *Clinical nodal involvement* | *Scan*[c] *ratio* | *Scan*[d] *result* |
|---|---|---|---|---|---|---|
| *$^{131}$I-3E1.2* | | | | | | |
| 1 | Left | Ductal | 0/18 | No | 1.0:1.0 | TN |
| 2 | Right | Ductal stage IV | + | Yes | 2.0:1.0 | TP |
| 3 | Right | Fibroadenoma | – | No | 1.0:1.0 | TN |
| 4 | Right | Ductal | 0/11 | Yes | 1.0:1.0 | TN |
| 5 | Right | Ductal stage IV | + | Yes | 1.0:1.0 | FN |
| 6 | Left | Ductal | 1/20 | No | 1.0:1.0 | FN |
| 7 | Right | Ductal | 2/18 | Yes | 1.0:1.0 | FN |
| 8 | Left | Ductal | 4/26 | No | 1.0:1.0 | FN |
| 9 | Right | Ductal | 0/6 | Yes | 1.0:1.0 | TN |
| 10 | Right | Ductal | 6/12 | No | 3.0:1.0 | TP |
| 11 | Left | Ductal | 5/11 | Yes | 1.0:1.0 | FN |
| 12 | Left | Ductal | 0/19 | No | 1.8:1.0 | FP |
| 13 | Right | Ductal | 5/9 | No | 1.0:1.0 | FN |
| 14 | Right | Ductal | 5/18 | Yes | 1.2:1.0 | FN |
| 15 | Left | Ductal | 4/10 | Yes | 1.0:1.0 | FN |
| 16 | Left | Ductal | 0/14 | No | 1.8:1.0 | FP |
| 17 | Left | Neuro-endocrine | 0 | No | 1.4:1.0 | TN |
| 18 | Right | Lobular | 0/8 | No | <1.0:1.0 | TN |
| 19 | Right | Ductal | 1/13 | No | 1.0:1.0 | FN |
| 20 | Right | Mixed ductal and lobular | 3/12 | No | 1.5:1.0 | TP |
| 21 | Right | Ductal | 0/10 | Yes | 2.6:1.0 | FP |
| 22 | Left | Ductal | 4/16 | Yes | 1.0:1.0 | FN |
| *$^{131}$I-RCC-1* | | | | | | |
| 23 | Left | Fibroadenoma | – | Yes | 1.0:1.0 | TN |
| 24 | Right | Ductal | 0/12 | Yes | 1.0:1.0 | TN |
| 25 | Left | Ductal | 1/14 | No | 2.0:1.0 | TP |
| 26 | Right | Ductal | 2/22 | No | 1.0:1.0 | FN |
| 27 | Right | Ductal | 0/15 | Yes | 1.5:1.0 | FP |
| 28 | Left | Lobular | 2/3 | No | 2.2:1.0 | TP |
| 29 | Right | Carcinoma *in situ* | 0/13 | No | 1.0:1.0 | TN |
| 30 | Right | Ductal | 3/13 | No | <1.0:1.0 | FN |
| 31 | Left | Ductal stage IV | + | Yes | 1.1:1.0 | FN |
| 32 | Left | Ductal | 14/21 | Yes | 3.0:1.0 | TP |
| 33 | Left | Fibroadenoma | – | No | 1.9:1.0 | FP |
| 34 | Right | Ductal | 0/16 | No | 1.4:1.0 | TN |
| 35 | Right | Lobular | 14/22 | Yes | 2.7:1.0 | TP |
| 36 | Right | Ductal | 0/14 | No | 1.2:1.0 | TN |
| 37 | Right | Lobular | 0 | Yes | <1.0:1.0 | TN |
| 38 | Right | Fibroadenoma | – | No | 2.0:1.0 | FP |
| 39 | Left | Ductal | 4/8 | Yes | 1.0:1.0 | FN |
| 40 | Right | Ductal | 0/14 | Yes | 2.0:1.0 | FP |

[a]All patients had breast carcinoma, except patients 3, 23, 33 and 38, who had fibroadenoma, and patient 17, who had malignant neuroendocrine tumour; [b]Nodal metastases expressed as the number of involved nodes/total number of nodes recovered from axillary dissection specimen; 0 indicates no nodal metastases but the total number of lymph nodes examined not specified; + indicates nodal metastases as assessed by clinical examination and fine needle aspiration cytology in patients with stage IV breast cancer; – indicates that axillary dissection was not performed; [c]Scan ratio expressed as the ratio of background-subtracted radioactive count of the axilla ipsilateral to breast tumour to that of contralateral axilla. A ratio $\geqslant$ 1.5:1.0 was regarded as indicative of lymph node metastases; [d]TP, true positive; FP, false positive; TN, true negative; FN, false negative.

**Table II** Comparison of immunoscintigraphy with clinical and pathological assessment of the axillae in patients with suspected breast cancer

| *Pathological*[a] *assessment* | *Immunoscintigraphy*[b] *+ve* | *Clinical assessment*[c] *+ve* | *Immunoscintigraphy +ve and/or clinical +ve* |
|---|---|---|---|
| $^{131}$*I-3E1.2* | | | |
| Node +ve | 3/13 (23%) | 7/13 (54%) | 9/13 (69%) |
| Node −ve | 3/9 (33%) | 3/9 (33%) | 5/9 (56%) |
| $^{131}$*I-RCC-1* | | | |
| Node +ve | 4/8 (50%) | 4/8 (50%) | 6/8 (75%) |
| Node −ve | 4/10 (40%) | 5/10 (50%) | 7/10 (70%) |

[a]Node +ve implies $\geqslant 1$ nodal metastases; node −ve implies no nodal metastases, as confirmed by histological or cytological examination; [b]+ve implies scan ratio of axilla of interest to contralateral axilla of $\geqslant 1.5:1.0$, which indicates the presence of lymph node metastases; [c]+ve implies palpable axillary lymph nodes felt to contain tumour deposits on clinical assessment.

### *Immunoscintigraphy with $^{131}$I-RCC-1 (IgG 2a)*

Patients 23–40 in Table I received $^{131}$I-RCC-1 and correct prediction of the axillary lymph node status by the scan was obtained in 56% (10/18) of the patients, comparable with preoperative clinical assessment (50% or 9/18) (Table II). Eight of the patients had histologically proven axillary lymph node metastases: 4/8 (50%) patients were detected by the scan and 4/8 (50%) patients by clinical examination. Ten of the 18 patients did not have lymph node metastases: a negative scan was obtained in 6/10 (60%) of the patients, and in 5/10 (50%) patients the axillae were considered not involved by metastases on clinical examination (Table I).

### *Illustrative cases*

Some examples of representative scintigraphy images are described in more detail below.

Patient 9 was considered to have involved axillary lymph nodes on clinical assessment but the scintigraphy images showed equal uptake of $^{131}$I-3E1.2 in both axillae with a ratio of background subtracted count of 1.0:1.0 between the two axillae (Figure 1). Histology of the six lymph nodes recovered showed reactive hyperplasia with no metastases.

Patient 10 was considered not to have involved axillary lymph nodes clinically but immunoscintigraphy with $^{131}$I-3E1.2 showed preferential uptake of radioactivity in the right axilla (ipsilateral to the side of breast cancer) with a scan ratio of 3.0:1.0 between right and left axillae (Figure 2). Subsequent histology of the axillary dissection specimen showed the presence of metastases in 6/12 lymph nodes.

Patient 32 with left sided breast cancer was considered to have axillary lymph node involvement clinically and to have scintigraphy images suggestive of lymph node metastases (scan ratio of left axilla to right axilla = 3.0:1.0); histology confirmed the presence of metastases in 14/21 lymph nodes (Figure 3).

Despite many scintigraphy images showing good preferential localisation in axillae with lymph node metastases, non-specific uptake of radiolabelled antibody in contralateral normal axilla by presumably normal lymph nodes was a major problem, making interpretation of the scintigraphy images difficult (Figure 4). This 'non-specific' uptake appeared to be more prominent with IgM antibody (3E1.2).

### *Biodistribution data*

In 40 patients the fraction of monoclonal antibody retained in the nodes (*F*), calculated as a percentage of the actual injected dose, was determined using formulae 1, 2 and 3 based on the gamma-camera count rates. These results are summarised in Table III and show that node positive axillae appeared to have a higher mean nodal uptake of radiolabelled RCC-1 (IgG) than those with negative nodes (1.3:1.0), although there was some overlap between the positive and negative axillae; however, such differences were not apparent with $^{131}$I-3E1.2 (IgM).

**Figure 1** Scintigraphic image of the anterior chest including both axillae in a patient with right breast cancer and palpably enlarged right axillary lymph nodes – none of the lymph nodes contained tumour deposits on histology. The respective regions of interest were as follows: A, right axilla; B, background region adjacent to right axillary region; C, left axilla and D, background region adjacent to left axillary region. Scan ratio of right to left = 1.0:1.0.

**Figure 2** Scintigraphic image showing an increased uptake of radiolabelled antibody in right axilla (scan ratio of right to left = 2.0:1.0) which corresponded to the presence of lymph node metastases.

**Figure 3** Scintigraphic image of anterior chest in a patient with left breast cancer and left axillary lymph node metastases. Scan ratio of left to right = 3.0:1.0.

**Figure 4** Scintigraphic image in a patient who received $^{131}$I-3E1.2. There was equally significant uptake of radioactivity in both right (R) and left (L) axillae (scan ratio = 1.0:1.0) although the patient had breast cancer and histologically proven lymph node metastases on the right side only. The uptake of radioactivity by thyroid (T) was noted despite prophylactic thyroid suppression with potassium iodide and sodium perchlorate.

**Table III** Nodal uptake (%) of $^{131}$I-3E1.2 or $^{131}$I-RCC-1 in axillae with and without lymph node metastases

| | *Mean nodal uptake (±s.d.) as % of injected dose*[b] | |
|---|---|---|
| *Pathology*[a] | $^{131}$*I-3E1.2* | $^{131}$*I-RCC-1* |
| Positive axilla | 4.8±1.6% | 5.6±2.0% |
| Negative axilla | 4.4±1.0% | 4.3±0.8% |

[a]Positive axilla implies ⩾1 lymph node metastases; negative implies there was no lymph node metastases, as assessed by histological or cytological examination; [b]Mean nodal uptake as calculated using formulae 1, 2 and 3 in the text; s.d. = standard deviation.

### Discussion

Currently the best prognostic factor in breast cancer, in the absence of distant dissemination, is the involvement of ipsilateral axillary lymph nodes. This is also the main indicator for the need for adjuvant therapy. As the clinical assessment of axillae is unreliable, the accurate detection of axillary lymph node metastases will usually require axillary dissection, a procedure which has an associated morbidity. The use of radiolabelled antibodies for the detection of lymph node metastases is therefore an attractive concept which, if sufficiently sensitive and specific, may replace surgical axillary dissection. However, despite recent reports on immunoscintigraphy (Epenetos, 1985; Thompson *et al.*, 1984), there are few prospective studies of immunoscintigraphy for the detection of axillary lymph node metastases and to relate findings from the scintigraphy with histology of the lymph nodes.

We now report a study of immunoscintigraphy in 40 patients. An important feature was the quantitative analysis of the scintigraphy images by obtaining a ratio of background-subtracted radioactive count of either axilla. This added objectivity to the interpretation of the scintigraphy images, compared to the conventional visual interpretation. It was found that a ratio ⩾1.5:1.0 between the axilla of interest and the normal axilla was significant and this was selected because at and above that ratio there was an obvious visual difference between the scintigraphy images of the axillae and, furthermore, it gave optimal accuracy in the detection of lymph node metastases in the study.

This study shows that immunoscintigraphy can localise lymph node metastases in a proportion of patients with breast cancer. The overall sensitivity in the detection of lymph node metastases was 33% (7/21) by immunoscintigraphy (23% with $^{131}$I-3E1.2 and 50% with $^{131}$I-RCC-1) and 52% (11/21) by clinical examination; the overall specificity was 63% by immunoscintigraphy (67% with $^{131}$I-3E1.2 and 60% with $^{131}$I-RCC-1) and 58% by clinical examination. While the specificity of immunoscintigraphy with $^{131}$I-3E1.2 was comparable with $^{131}$I-RCC-1 ($P = 0.5$), there was an improvement in sensitivity in the detection of lymph node metastases with IgG (RCC-1) antibody when compared with IgM (3E1.2) antibody, although the differences in sensitivity were not statistically significant, mainly because of the small number of patients studied ($P = 0.2$) (Table II). In addition, there were cases when immunoscintigraphy detected axillary lymph node metastases not suspected by clinical examination. The overall accuracy of immunoscintigraphy using $^{131}$I-RCC-1 (56% or 10/18) was not much superior to preoperative clinical assessment (50% or 9/18). If both modalities of assessment (clinical assessment and immunoscintigraphy) were combined, in that either abnormal finding was regarded as significant, there was an improvement in sensitivity in the detection of lymph node metastases but with a concomitant deterioration in the specificity (Table II).

The presence of tumour-associated antigens recognised by monoclonal antibodies 3E1.2 and RCC-1 was demonstrated in the lymph node of some patients by immunoperoxidase staining (data not shown) and high nodal uptake of radioactivity in some axillae with lymph node metastases compared with normal axillae was noted (patients 10 and 32). However, the concentration of radiolabelled antibody in the axillae, calculated using formulae 1, 2 and 3, indicated that when $^{131}$I-RCC-1 was used, the fraction of injected dose accumulated in the axillae with involved nodes (~5.6%) was only slightly higher than in those with uninvolved nodes (~4.3%) although there was some overlap between the positive and negative axillae. There was no difference when $^{131}$I-3E1.2 was administered (4.8% *vs.* 4.4%). The correlation of the uptake of radiolabelled antibody by involved lymph nodes with their antigen content as assessed by immunoperoxidase staining would be of interest but was not performed in each case because of the practical difficulty of obtaining and identifying fresh lymph nodes in each case (RCC-1 antibody only reacts with fresh tissue sections).

A major problem identified was the non-specific uptake of radiolabelled antibody by normal lymph nodes, especially when IgM antibody was administered. This made interpretation of the scintigraphic image difficult, even with the quantitative analysis to obtain a ratio of background-subtracted radioactive count between the axilla of interest and the normal contralateral axilla. Thus, the presence of reactive lymph nodes in the axilla, on the side of breast cancer, can lead to the uptake of radiolabelled antibody leading to false positive scintigraphic images, while the non-specific uptake of radioactive material in the normal contralateral axilla could also lead to false negative interpretation of scintigraphic images. This phenomenon has also been described by other investigators (Epenetos, 1985; Nelp *et al.*, 1987). It is likely that this may be due, in part, to binding through the Fc portion of the mouse immunoglobulin. The large size of the IgM antibody probably caused significantly more non-specific binding than the smaller IgG antibody. However, when radiolabelled F(ab')2 fragments of RCC-1 antibody were used for immunoscintigraphy, there was equally significant non-specific uptake of radiolabelled antibody fragment by normal axilla (data not shown). Further larger studies with antibody fragments to avoid Fc binding or with other measures which may saturate non-specific binding sites of normal lymph nodes, such as the addition to radiolabelled antibody of excess irrelevant antibody of the same Ig isotype, are currently in progress.

Although the results of immunoscintigraphy in this study are of interest, and indicate that it may be a useful adjunct to preoperative clinical assessment, a major improvement in its accuracy is needed before axillary dissection and histological examination can be replaced as the standard method of staging axilla in breast cancer. However, immunoscintigraphy is simple and safe, and it is more specific than colloid lymphoscintigraphy (Gasparini *et al.*, 1987), a technique in which radiolabelled particles are injected subcutaneously and are taken up by macrophages in the draining lymph nodes. Subcutaneous immunoscintigraphy is also more efficient than the intravenous route of immunoscintigraphy when the aim is to deliver radiolabelled antibody to regional lymph nodes (Bunn *et al.*, 1984; Keenan *et al.*, 1987). The results of this prospective study fell short of the previous results (Thompson *et al.*, 1984) and indicated that the one IgG antibody used was preferable to an IgM antibody, but whether this holds true for other antibodies remains to be seen. However, the major problem identified is the so-called 'non-specific' uptake of mouse immunoglobulin by the draining lymph nodes. However, while this is an annoying problem in immunoscintigraphy, we suspect greater clinical problems would result if the lymph nodes were not able to remove up to 1 mg of foreign protein, i.e. the 'non-specific' uptake is normal. How this is to be overcome is now a major problem in immunoscintigraphy, particularly when examining the regional lymph nodes. This study clearly demonstrates that antibodies for tumour imaging must be assessed prospectively in large clinical studies and that monoclonal antibodies of the IgM class are not optimal for radioimmunolocalisation by lymphoscintigraphy.

The authors would like to thank the Nuclear Medicine and Anatomical Pathology departments of the Royal Melbourne Hospital for their assistance. We are also grateful to Toula Athanasiadis for her secretarial assistance and to Mr David Binn for his technical assistance.

### References

ARMITAGE, N.C., PERKINS, A.C., PIMM, M.V., FARRANDS, P.A., BALDWIN, R.W. & HARDCASTLE, J.D. (1984). The localization of an anti-tumour monoclonal antibody (791T/36) in gastrointestinal tumours. *Br. J. Surg.*, **71**, 407.

BEARHS, O.H. & MYERS, M.H. (1983). *American Joint Committee on Staging Manual for Staging of Cancer.* J.B. Lippincott: Philadelphia.

BERGVIST, L., STRAND, S.E. & PERSSON, B.R.R. (1983). Particle sizing and biokinetics of interstitial lymphoscintigraphic agents. *Semin. Nucl. Med.*, **13**, 9.

BRADWELL, A.R., FAIRWEATHER, D.S., DYKES, P.W., KELLING, A., VAUGHAN, A. & TAYLOR, J. (1985). Limiting factors in the localisation of tumours with radiolabelled antibodies. *Immunol. Today*, **6**, 163.

BUNN, P.A., CARRASQUILLO, J.A., KEENAN, A.M. & 7 others (1984). Imaging of T-cell lymphoma by radiolabelled monoclonal antibody. *Lancet*, **ii**, 1219.

DELAND, F.H., KIM, E.E., CORGAN, R.L. & 5 others (1979). Axillary lymphoscintigraphy by radioimmunodetection of carcinoembryonic antigen in breast cancer. *J. Nucl. Med.*, **20**, 1243.

EPENETOS, A.A. (1985). Antibody guided lymphangiography in the staging of cervical cancer. *Br. J. Cancer*, **51**, 805.

GASPARINI, M., ANDREOLI, C., RODARI, A., COSTA, A. & BURAGGI, G.L. (1987). Lack of efficacy of lymphoscintigraphy in detecting axillary lymph node metastases from breast cancer. *Eur. J. Cancer Clin. Oncol.*, **23**, 475.

GREENWOOD, F.C., INNTER, W.M. & GLOVER, J.S. (1963). The preparation of $^{131}$I-labelled human growth hormone of high specific radioactivity. *Biochem. J.*, **89**, 114.

KEENAN, A.M., WEINSTEIN, J.N., MULSHINE, J.L. & 4 others (1987). Immunolymphoscintigraphy in patients with lymphoma after subcutaneous injection of indium-111-labelled T101 monoclonal antibody. *J. Nucl. Med.*, **28**, 42.

LEAK, L.V. (1971). Studies on the permeability of lymphatic capillaries. *J. Cell Biol.*, **50**, 300.

LEYDEN, M.L., THOMPSON, C.H., LICHTENSTEIN, M. & 4 others (1986). Visualisation of metastases from colon carcinoma using an Iodine 131-radiolabelled monoclonal antibody. *Cancer*, **57**, 1135.

MACH, J.P., BUCHEGGER, F., GIRARDET, C. & 7 others (1981). Use of radiolabelled monoclonal anti-CEA antibodies for the detection of human carcinomas by external photoscanning and tomoscintigraphy. *Immunol. Today*, **2**, 239.

MARKWELL, M.A.K. (1982). A new solid state reagent to iodinate proteins. *Ann. Biochem.*, **125**, 427.

NELP, W.B., EARY, J.F. & JONES, R.F. (1987). Preliminary studies of monoclonal antibody lymphoscintigraphy in malignant melanoma. *J. Nucl. Med.*, **28**, 34.

PARISH, C.R. & MCKENZIE, I.F.C. (1978). A sensitive rosetting method for detecting subpopulations of lymphocytes which react with alloantisera. *J. Immunol. Meth.*, **20**, 173.

RAINSBURY, R.M. (1984). The localization of human breast carcinomas by radiolabelled monoclonal antibodies. *Br. J. Surg.*, **71**, 805.

ROCKOFF, S.D., GOODENOUGH, D.S. & MCINTIRE, K.R. (1980). Theoretical limitations in the immunodiagnostic imaging of cancer with computer tomography and nuclear scanning. *Cancer Res.*, **50**, 3054.

SCHROFF, R.W., FOON, K.A., BEATTY, S.M., OLDHAM, R.K. & MORGAN, A.C. (1985). Human anti-murine immunoglobulin responses in patients receiving monoclonal antibody therapy. *Cancer Res.*, **45**, 879.

SMEDLEY, H.M., FINAN, P., LENNOX, E.S. & 4 others (1983). Localisation of metastatic carcinoma by a radiolabelled monoclonal antibody. *Br. J. Cancer*, **47**, 253.

STACKER, S.A., THOMPSON, C.H., RIGLER, C. & MCKENZIE, I.F.C. (1985). A new breast carcinoma antigen defined by a monoclonal antibody. *J. Natl Cancer Inst.*, **75**, 801.

THOMPSON, C.H., JONES, S.L., WHITEHEAD, R.H. & MCKENZIE, I.F.C. (1983). A human breast tissue-associated antigen detected by a monoclonal antibody. *J. Natl Cancer Inst.*, **70**, 409.

THOMPSON, C.H., LICHTENSTEIN, M., STACKER, S.A. & 4 others (1984). Immunoscintigraphy for detection of lymph node metastases from breast cancer. *Lancet*, **ii**, 1245.

TJANDRA, J.J., BUSMANIS, I., RUSSELL, I.S.R., COLLINS, J.P., REED, R.G. & MCKENZIE, I.F.C. (1988). The association of mammary serum antigen (MSA) with the histopathological findings in localized breast cancer. *Br. J. Cancer*, **58**, 815.

WEINSTEIN, J.N., PARKER, R.J., KEENAN, A.M. & 3 others (1982). Monoclonal antibodies in the lymphatics: toward the diagnosis and therapy of tumour metastases. *Science*, **218**, 1334.

WEINSTEIN, J.N., STELLER, M.A., KEENAN, A.M. & 6 others (1983). Monoclonal antibodies in the lymphatics: selective delivery to lymph node metastases of a solid tumour. *Science*, **22**, 423.

WEINSTEIN, J.N., STELLER, M.A., COVELL, D.G. & 4 others (1984). Monoclonal antitumour antibodies in the lymphatics. *Cancer Treat. Rep.*, **68**, 257.

WILLIAM, M.R., PERKINS, A.C., CAMPBELL, F.C. & 5 others (1984). The use of monoclonal antibody 791 T/36 in the immunoscintigraphy of primary and metastatic carcinoma of the breast. *Clin. Oncol.*, **10**, 375.


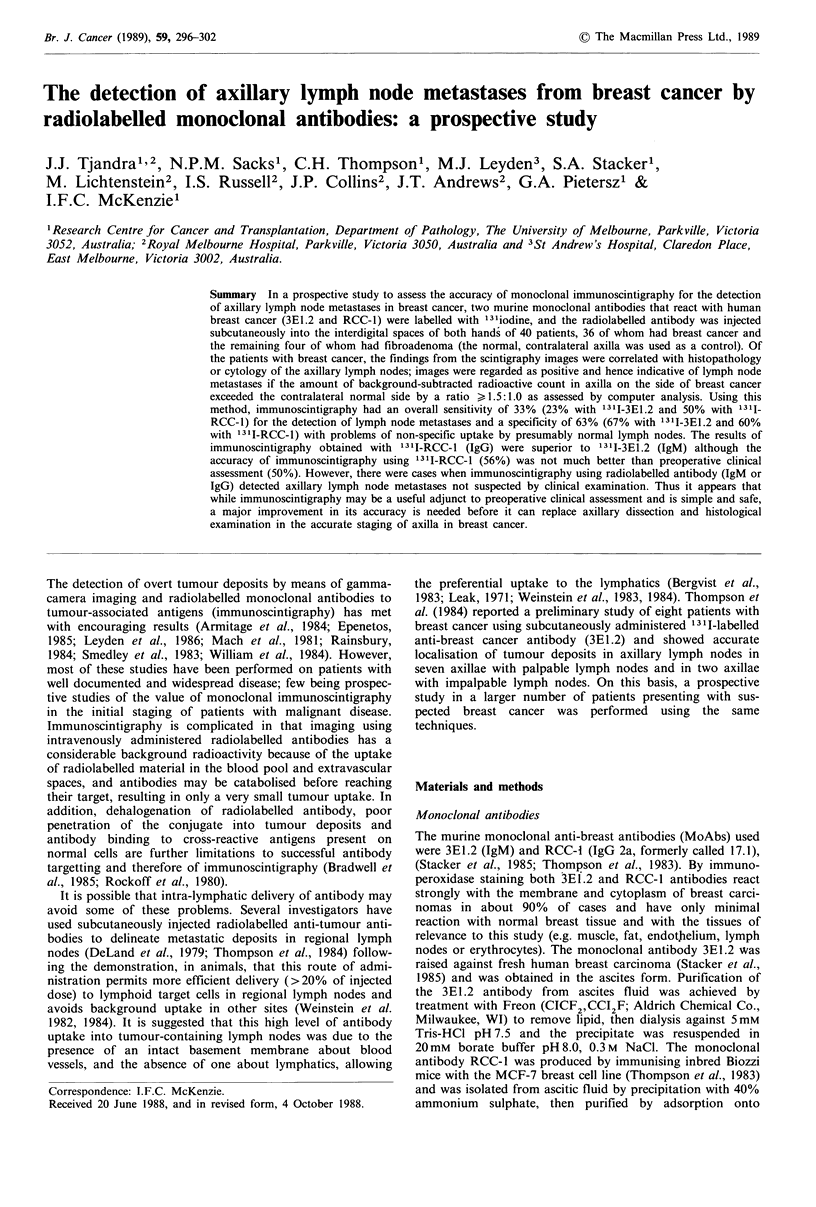

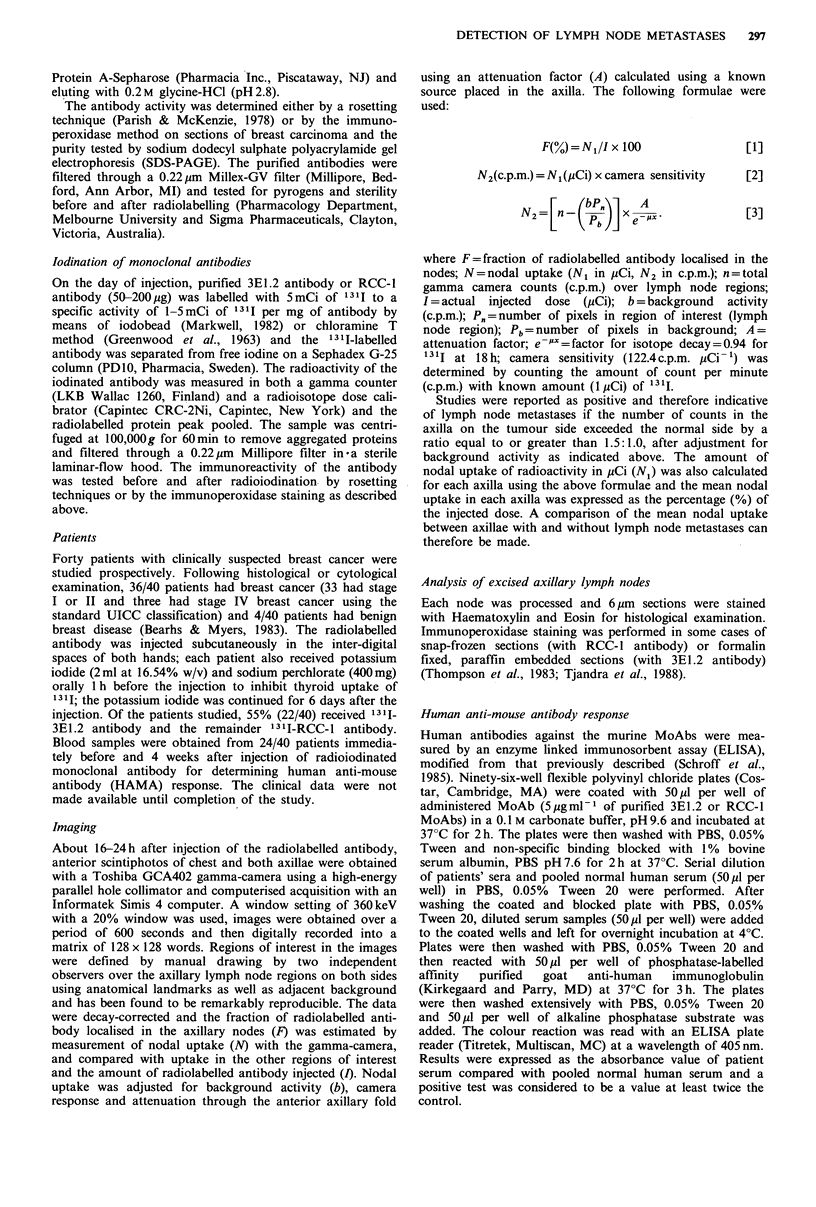

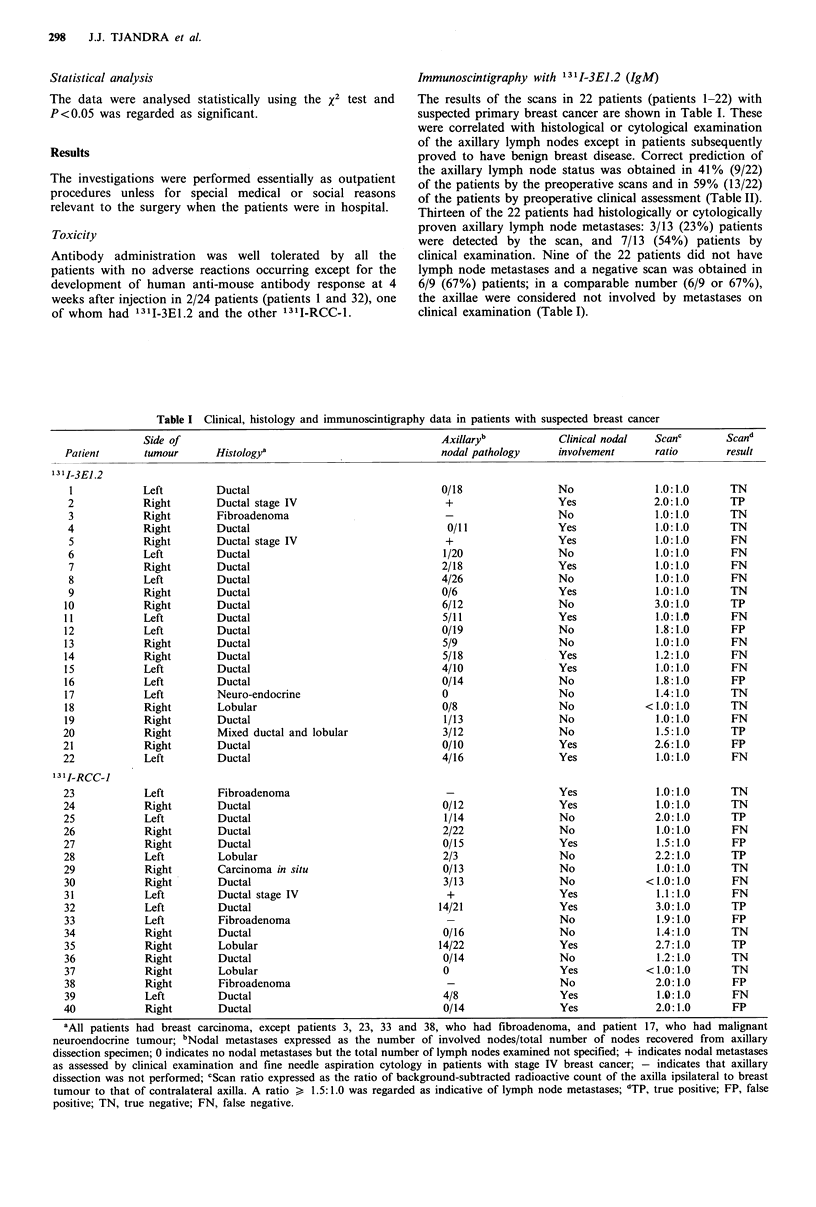

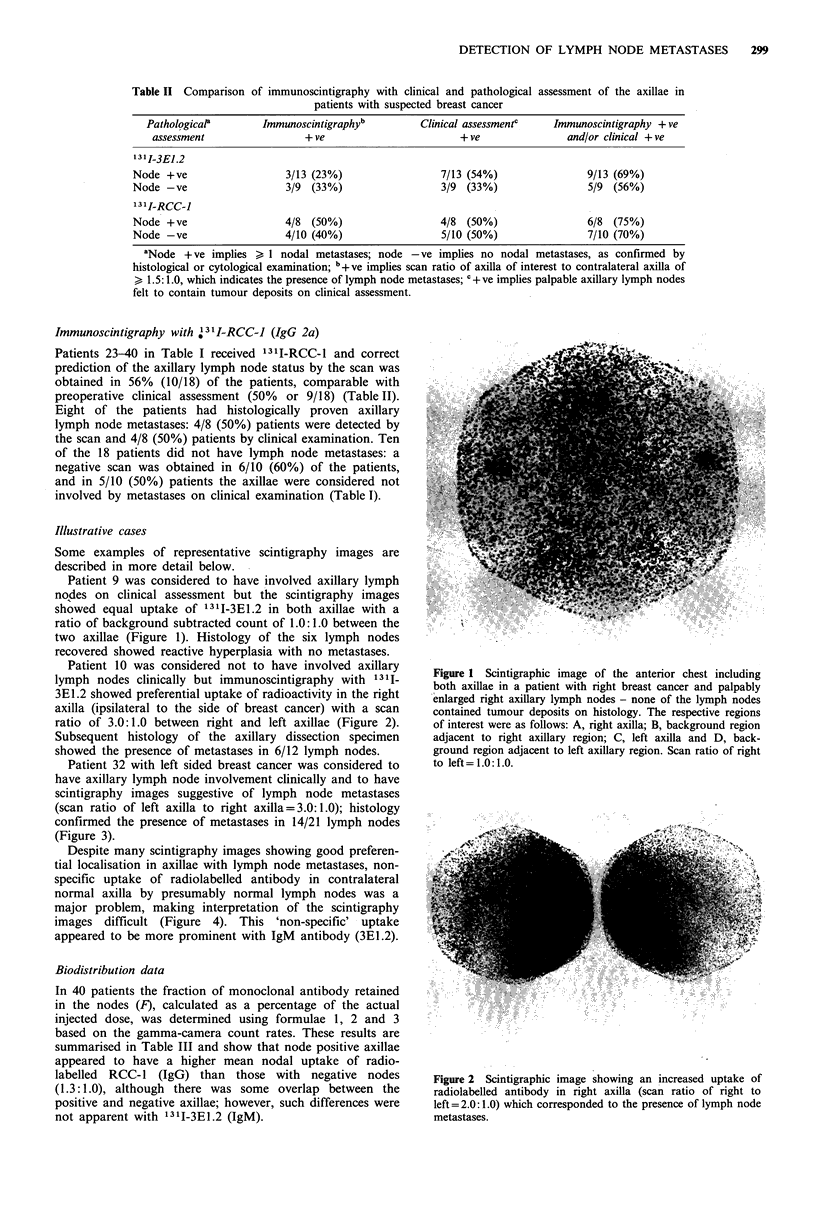

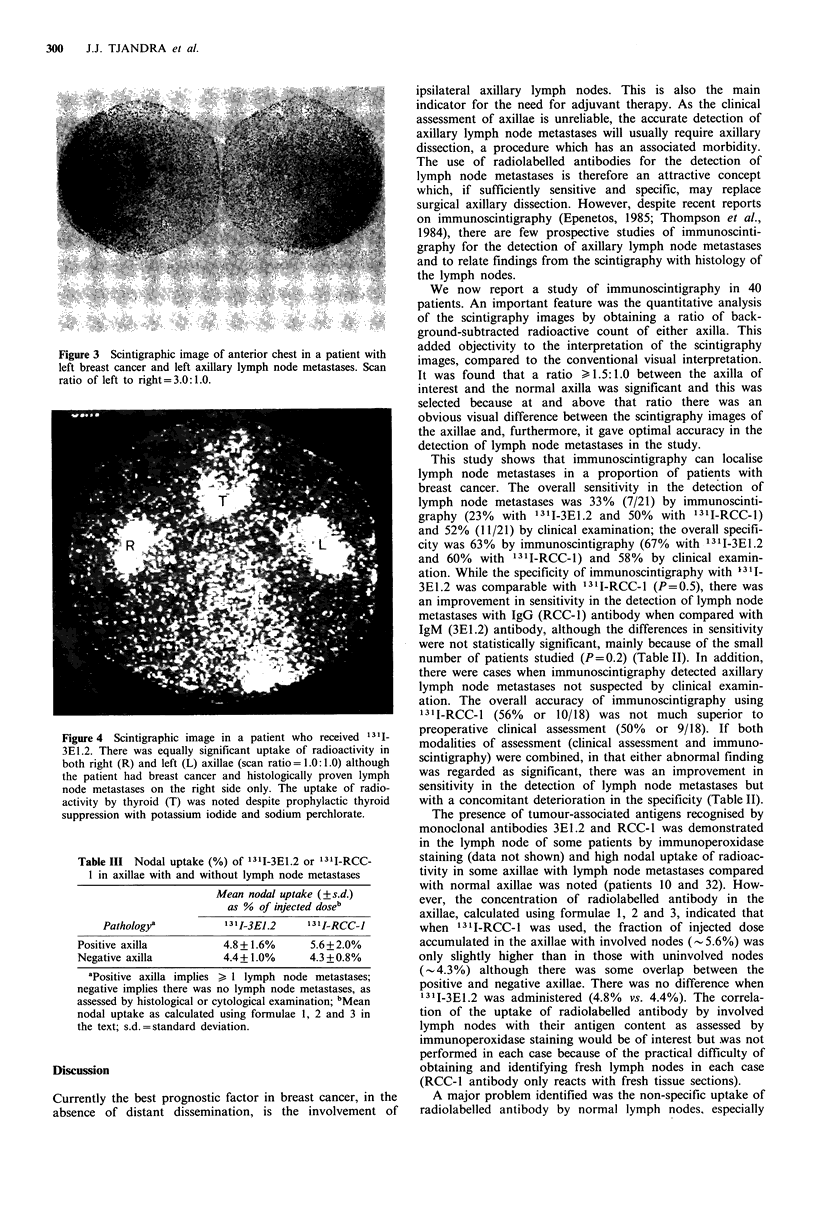

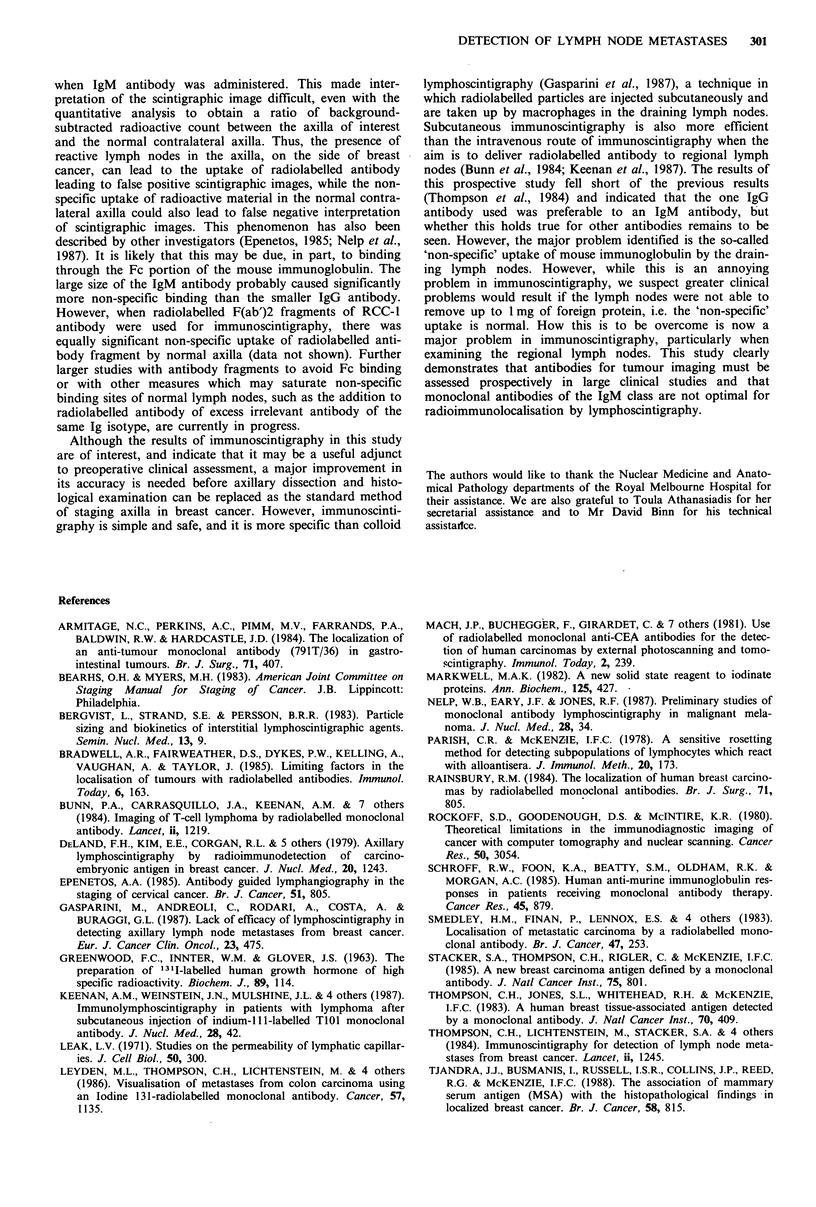

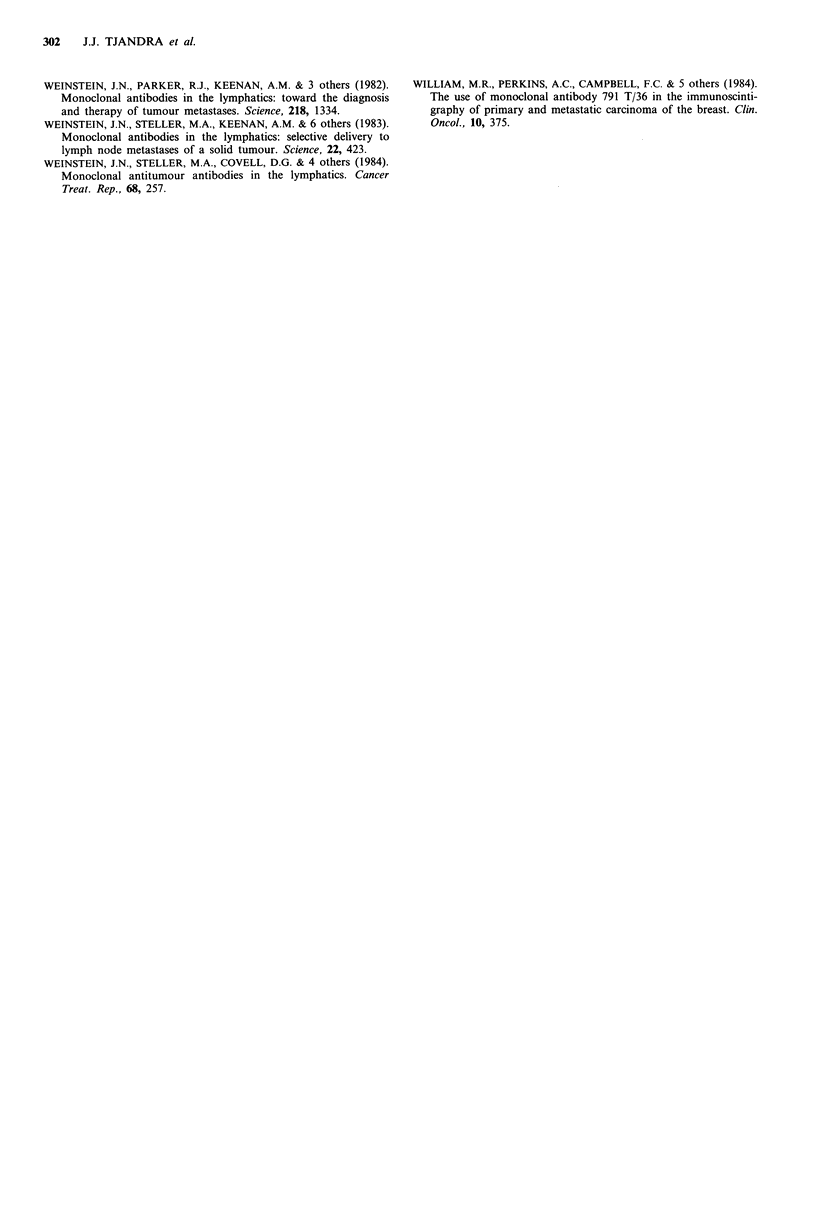


## References

[OCR_00824] Armitage N. C., Perkins A. C., Pimm M. V., Farrands P. A., Baldwin R. W., Hardcastle J. D. (1984). The localization of an anti-tumour monoclonal antibody (791T/36) in gastrointestinal tumours.. Br J Surg.

[OCR_00835] Bergqvist L., Strand S. E., Persson B. R. (1983). Particle sizing and biokinetics of interstitial lymphoscintigraphic agents.. Semin Nucl Med.

[OCR_00846] Bunn P. A., Carrasquillo J. A., Keenan A. M., Schroff R. W., Foon K. A., Hsu S. M., Gazdar A. F., Reynolds J. C., Perentesis P., Larson S. M. (1984). Imaging of T-cell lymphoma by radiolabelled monoclonal antibody.. Lancet.

[OCR_00856] Epenetos A. A. (1985). Antibody guided lymphangiography in the staging of cervical cancer.. Br J Cancer.

[OCR_00866] GREENWOOD F. C., HUNTER W. M., GLOVER J. S. (1963). THE PREPARATION OF I-131-LABELLED HUMAN GROWTH HORMONE OF HIGH SPECIFIC RADIOACTIVITY.. Biochem J.

[OCR_00860] Gasparini M., Andreoli C., Rodari A., Costa A., Buraggi G. L. (1987). Lack of efficacy of lymphoscintigraphy in detecting axillary lymph node metastases from breast cancer.. Eur J Cancer Clin Oncol.

[OCR_00851] Kim E. E., Corgan R. L., Casper S., Primus F. J., Spremulli E., Estes N., Goldenberg D. M. (1979). Axillary lymphoscintigraphy by radioimmunodetection of carcinoembryonic antigen in breast cancer.. J Nucl Med.

[OCR_00877] Leak L. V. (1971). Studies on the permeability of lymphatic capillaries.. J Cell Biol.

[OCR_00881] Leyden M. J., Thompson C. H., Lichtenstein M., Andrews J. T., Sullivan J. R., Zalcberg J. R., McKenzie I. F. (1986). Visualization of metastases from colon carcinoma using an iodine 131-radiolabeled monoclonal antibody.. Cancer.

[OCR_00893] Markwell M. A. (1982). A new solid-state reagent to iodinate proteins. I. Conditions for the efficient labeling of antiserum.. Anal Biochem.

[OCR_00897] Nelp W. B., Eary J. F., Jones R. F., Hellstrom K. E., Hellstrom I., Beaumier P. L., Krohn K. A. (1987). Preliminary studies of monoclonal antibody lymphoscintigraphy in malignant melanoma.. J Nucl Med.

[OCR_00902] Parish C. R., McKenzie I. F. (1978). A sensitive rosetting method for detecting subpopulations of lymphocytes which react with alloantisera.. J Immunol Methods.

[OCR_00907] Rainsbury R. M. (1984). The localization of human breast carcinomas by radiolabelled monoclonal antibodies.. Br J Surg.

[OCR_00912] Rockoff S. D., Goodenough D. J., McIntire K. R. (1980). Theoretical limitations in the immunodiagnostic imaging of cancer with computed tomography and nuclear scanning.. Cancer Res.

[OCR_00918] Schroff R. W., Foon K. A., Beatty S. M., Oldham R. K., Morgan A. C. (1985). Human anti-murine immunoglobulin responses in patients receiving monoclonal antibody therapy.. Cancer Res.

[OCR_00924] Smedley H. M., Finan P., Lennox E. S., Ritson A., Takei F., Wraight P., Sikora K. (1983). Localisation of metastatic carcinoma by a radiolabelled monoclonal antibody.. Br J Cancer.

[OCR_00929] Stacker S. A., Thompson C., Riglar C., McKenzie I. F. (1985). A new breast carcinoma antigen defined by a monoclonal antibody.. J Natl Cancer Inst.

[OCR_00934] Thompson C. H., Jones S. L., Whitehead R. H., McKenzie I. F. (1983). A human breast tissue-associated antigen detected by a monoclonal antibody.. J Natl Cancer Inst.

[OCR_00939] Thompson C. H., Lichtenstein M., Stacker S. A., Leyden M. J., Salehi N., Andrews J. T., McKenzie I. F. (1984). Immunoscintigraphy for detection of lymph node metastases from breast cancer.. Lancet.

[OCR_00944] Tjandra J. J., Busmanis I., Russell I. S., Collins J. P., Reed R. G., McKenzie I. F. (1988). The association of mammary serum antigen (MSA) with the histopathological findings in localised breast cancer.. Br J Cancer.

[OCR_00962] Weinstein J. N., Steller M. A., Covell D. G., Holton O. D., Keenan A. M., Sieber S. M., Parker R. J. (1984). Monoclonal antitumor antibodies in the lymphatics.. Cancer Treat Rep.

[OCR_00952] Weinstein J. N., Steller M. A., Keenan A. M., Covell D. G., Key M. E., Sieber S. M., Oldham R. K., Hwang K. M., Parker R. J. (1983). Monoclonal antibodies in the lymphatics: selective delivery to lymph node metastases of a solid tumor.. Science.

[OCR_00967] Williams M. R., Perkins A. C., Campbell F. C., Pimm M. V., Hardy J. G., Wastie M. L., Blamey R. W., Baldwin R. W. (1984). The use of monoclonal antibody 791T/36 in the immunoscintigraphy of primary and metastatic carcinoma of the breast.. Clin Oncol.

